# Validation of a Finite Element Simulation for Predicting Individual Knee Joint Kinematics

**DOI:** 10.1109/OJEMB.2023.3258362

**Published:** 2023-03-16

**Authors:** Elin Theilen, Anna Rörich, Thomas Lange, Sebastian Bendak, Cora Huber, Hagen Schmal, Kaywan Izadpanah, Joachim Georgii

**Affiliations:** ^1^ Fraunhofer Institute for Digital Medicine MEVIS 201971 28359 Bremen Germany; ^2^ Division of Medical Physics, Department of Diagnostic and Interventional Radiology, Medical Center - University of Freiburg, Faculty of MedicineUniversity of Freiburg 9174 79104 Freiburg Germany; ^3^ Department of Orthopedic Surgery and Traumatology, Freiburg University HospitalAlbert-Ludwigs-University Freiburg 9174 79106 Freiburg Germany; ^4^ Stryker Leibinger GmbH & Co. KG 39139 79111 Freiburg im Breisgau Germany

**Keywords:** Finite element knee joint model, model validation, joint motion prediction, subject-specific kinematics, pneumatic loading device

## Abstract

*Goal:* We introduce an in-vivo validated finite element (FE) simulation approach for predicting individual knee joint kinematics. Our vision is to improve clinicians' understanding of the complex individual anatomy and potential pathologies to improve treatment and restore physiological joint kinematics. *Methods:* Our 3D FE modeling approach for individual human knee joints is based on segmentation of anatomical structures extracted from routine static magnetic resonance (MR) images. We validate the predictive abilities of our model using static MR images of the knees of eleven healthy volunteers in dedicated knee poses, which are achieved using a customized MR-compatible pneumatic loading device. *Results:* Our FE simulations reach an average translational accuracy of 2 mm and an average angular accuracy of 1$^\circ$ compared to the reference knee pose. *Conclusions:* Reaching high accuracy, our individual FE model can be used in the decision-making process to restore knee joint stability and functionality after various knee injuries.

## I. Introduction

The knee is one of the most complex joints in the human body and its kinematics is unique and defined by the individual anatomy [1]. Further, the knee is very susceptible to injury, especially for sporty, active people or elderly people suffering from degenerative processes of the musculoskeletal system. Common injuries are tears of the cruciate and collateral ligaments, defects of the articular cartilage, or injuries of the menisci. Moreover, misalignment of the leg or non-physiological loading can lead to cartilage degeneration and development of a joint arthrosis [2]. An informed analysis about the injury's severity and its impact on the joint kinematics are of great importance to avoid long-term consequences or secondary complications like osteoarthritis or re-tearing the ligament. To restore joint stability and patient-individual joint kinematics, an accurate and comprehensive analysis of the knee joint kinematics is required. Analysing the knee joint kinematics can therefore have a significant impact on patient's outcome and can thus be an important tool for deciding on the best therapeutic intervention for each individual patient. In today's clinical routine, static imaging techniques (e.g. X-ray or magnetic resonance (MR) imaging) are usually used for diagnosis. A standardised assessment of the individual (restored) joint kinematics is so far not achievable. Thus, we propose an in-vivo validated finite element (FE) modeling approach, which is able to predict the individual knee joint kinematics from a set of static MR images.

Dynamic 3D knee joint models based on the finite element method (FEM) are widely used in research to assess the individual biomechanics [3], [4], [5]. To incorporate the muscle activity, combined FE/multi body system models are used [6], [7], [8]. More advanced FE models incorporate active muscles in an inverse-dynamic simulation setup [9]. In most literature, the knee joint models are generic or built up only from one single subject and lack explicit clinical validation, see for instance the review article [10]. Reasons are a lack of appropriate in-vivo measuring methods, since optical tracking with skin markers, as used in [11], does not accurately reflect the underlying bone motion. Prediction of the individual knee joint kinematics is used to determine complications, implant failure or help increasing the longevity of implants [12], [13]. Furthermore, assessment of meniscal or ligament tears can be accomplished with this modeling approach [5], [14], [15]. In-vivo analysis of meniscal motion under internal and external rotation torque using a measurement setup including a loading device and MR imaging has been demonstrated recently [16].

We propose a passive FE-based motion model that can be built from static MR images and predict the knee kinematics based on the individual anatomy. So far, muscles have not been included in this model. Each individual knee joint model is semi-automatically derived from segmentation of anatomical structures extracted from MR images. Moreover, we validate the model using in-vivo measurements of eleven healthy volunteers. The model validation is done using static MR images in various well-defined joint positions (20$^\circ$ flexion, 5 Nm internal/external rotation torque), which are supported by a MR-compatible pneumatic loading device and real-time motion correction for high-resolution, low-artifact knee measurements [17].

## II. Materials and Methods

To build individual FE knee models, a semi-automatic model generation process has been developed. It is composed of the following steps: the anatomy of the human knee is manually segmented in the MR images and used for creating 3D surface models. The resulting geometries are processed to ensure suitability for a FE model. After the mesh generation, boundary and contact conditions are automatically detected and written into a simulation control file. This file is used in the open source software FEBio [18], [19] for non-linear implicit FE simulations.

### A. Geometry

**Fig. 1.**
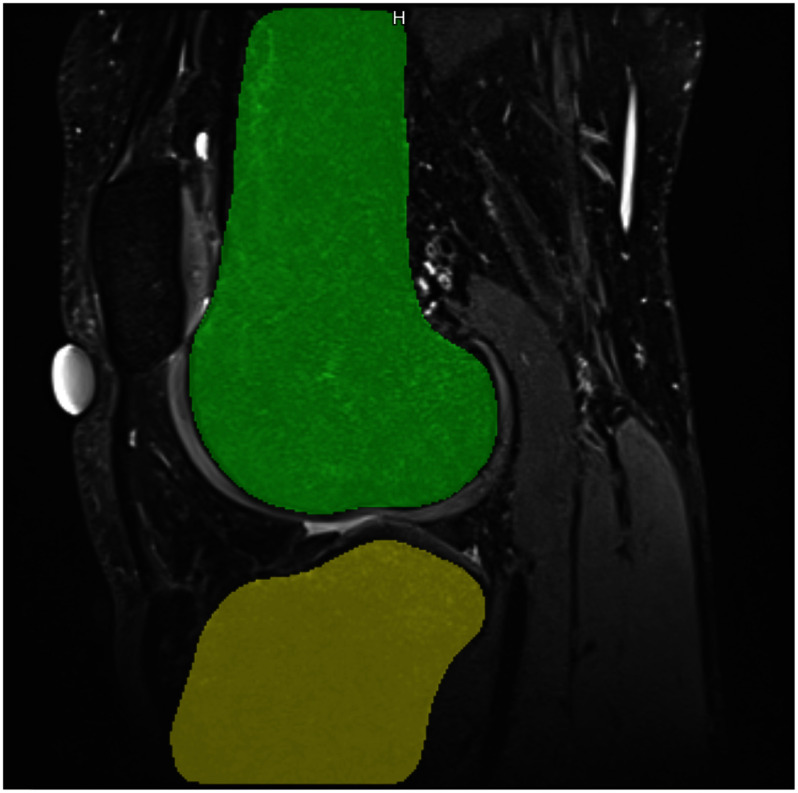
Sagittal view of the tibia and femur segmentation (in color) superimposed with the original MRI scan of a healthy human knee.

**Fig. 2.**
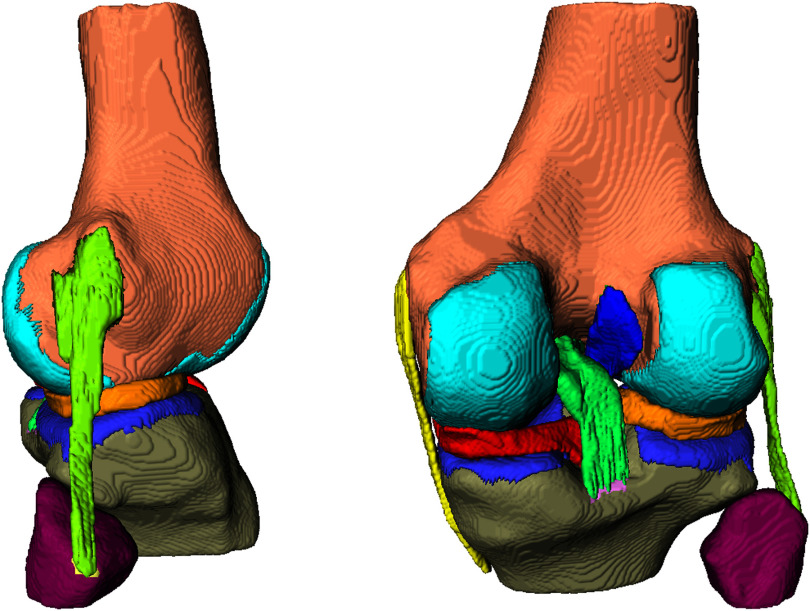
Overview of the anatomical structures considered in the FE model (lateral (left) and posterior view (right)): distal femur and proximal tibia with their corresponding articular cartilage, the proximal fibula, the cruciate and collateral ligaments, and the medial and lateral menisci.

For the development of individual knee models, eleven healthy volunteers (five women and six men, average age and standard deviation: $25\pm 2$ years) were selected. The examined knee had to be free of any previous surgery, chronic pain, relevant post trauma, signs of any degenerative changes or lesions on bone, cartilage, meniscus or ligaments. The study was approved by the ethics committee of the Albert-Ludwigs University Freiburg (Nr. 91/19 – 210696, 19 August 2021) and all volunteers gave written informed consent prior to participation. For the individual knee model, two high-resolution MR imaging data sets were acquired with the knee in full extension on a Magnetom Prisma 3 T system (Siemens Healthineers, Germany) using a dedicated Tx/Rx 15-channel knee coil. For delineation of bones, cartilages, tendons, and ligaments, a 3D turbo-spin echo (TSE) protocol with fat suppression based on adiabatic inversion recovery (SPAIR), a CAIPIRINHA acceleration factor of 2 and an isotropic resolution of 0.5 mm was used. Further sequence parameters were: field-of-view (FOV): 160 mm anterior-posterior (AP) × 144 mm right-left (RL) × 160 mm feet-head (FH), repetition time TR = 900 ms, echo time TE = 74 ms, readout bandwidth = 381 Hz/Px, readout direction = FH. The following main knee structures are segmented in the Prisma MR images: distal femur, proximal tibia and fibula and the corresponding articular cartilages, medial and lateral meniscus, anterior cruciate ligament (ACL), posterior cruciate ligament (PCL), medial collateral ligament (MCL), lateral collateral ligament (LCL). All structures have been manually segmented by clinical experts in the annotation platform SATORI (Segmentation and Annotation Tool for Radiomics and Deep Learning), such that automatic segmentation models can be trained on the annotated data in future. A description of SATORI and another use case thereof can be found in [20]. See Fig. 1 for exemplary segmentation of the distal femur and proximal tibia. An amalgamated model from the segmentation is visualized in 3D in Fig. 2.

**Fig. 3.**
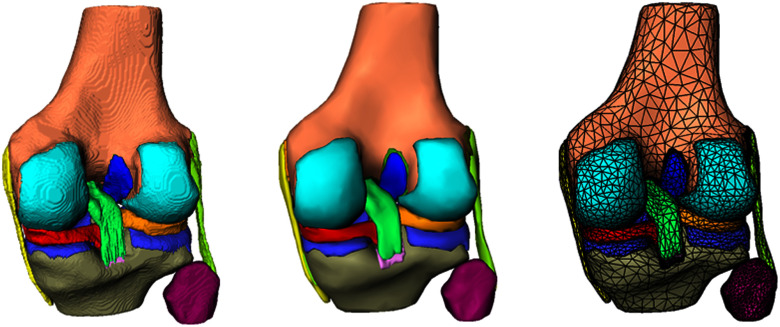
Processing pipeline of a 3-D knee joint model (from left to right): assemble segmentations, smoothing and cropping, final mesh generation.

For generating the FE model, thorough postprocessing of the segmented structures (masks) is required to be able to neatly define contact conditions. The developed automatic procedure ensures reasonable geometries and prevents the structures from overlapping through smoothing of the structures and cropping overlapping regions using boolean operations of the OpenVDB library [21]. The postprocessing has been implemented in the graphical prototyping platform MeVisLab [22], a modular framework for medical image processing. Subsequently, tetrahedral meshes are automatically generated using TetGen [23]. Note that low order element types were used to keep the automation process simple. The mesh resolution parameters have been chosen such that we obtain reasonable prediction accuracy at the lowest computational costs. Fig. 3 shows the processing pipeline and the mesh generation for an exemplary 3D FE knee model. Postprocessing and mesh generation takes only a few minutes.

### B. Material Properties and Strains

*Bones* are considered to be rigid and not deformable. Thus, distal femur, proximal tibia and fibula are represented by their surface meshes.

*Articular cartilages* are assumed to consist of Neo-Hookean material, i.e., a compressible material with non-linear stress-strain behaviour. The material characteristics are derived from the hyperelastic strain-energy function

\begin{equation*}
W = W (I_{1}, J, E, \nu) \tag{1}
\end{equation*}

that depends on the first invariant of the Cauchy-Green deformation tensor $I_{1}$, the determinant of the deformation gradient tensor $J$, Young's modulus $E$ and Poisson's ratio $\nu$ [24]. Young's modulus and Poisson's ratio are chosen according to Table I.

**TABLE I:** Neo-Hookean Material Parameters for Cartilage Modelling

Young's modulus $E$	10 MPa
Poisson's ratio $\nu$	0.25

*Menisci* are modelled using Fung elasticity with orthotropic symmetry [25]. The strain-energy for this hyperelastic constitutive relation consists of a distortional (isochoric) and a dilatational (volumetric) part $V$ and $U$:

\begin{equation*}
W = \frac{c}{2} V + U \tag{2}
\end{equation*}

Here, $c$ is a material coefficient with units of stress, $V = V(E_{1}, E_{2}, E_{3}, G_{12}, G_{23}, G_{31}, \nu _{12}, \nu _{23}, \nu _{31})$ depends on Young's moduli $E_{i}$, shear moduli $G_{ij}$ and Poisson's ratios $\nu _{ij}$, while $U = U(k)$ depends on the bulk modulus $k$, for more details cf. [26]. Specific values used for menisci modelling are given in Table II.

**TABLE II:** Orthotropic Fung Material Parameters [MPa] for Menisci Modelling

$E_{1}$	$E_{2}$	$E_{3}$	$G_{12}$	$G_{23}$	$G_{31}$	$\nu _{12}$	$\nu _{23}$	$\nu _{31}$	$c$	$k$
120	20	20	57.7	8.33	57.7	0.3	0.2	0.3	1	10

The attachments of the meniscal horn on the tibial plateau are represented by linear springs, inspired by [3].

*Ligaments* are described applying transversely isotropic material using a fully-coupled formulation featuring initial strains taken from available literature [27], [28], [29]. The Mooney-Rivlin model represents a material with a single preferred fibre direction. The elastic response is assumed to arise from the resistance of the fibre family and an isotropic matrix. Hence, the corresponding strain-energy function combines the strain energy $F_{1}$ of the isotropic ground matrix, the fibre strain energy $F_{2}$, and a volumetric part:

\begin{equation*}
W = F_{1} + F_{2} + \frac{k}{2} [\ln J]^{2} \tag{3}
\end{equation*}

Here, $F_{1} = F_{1}(C_{1}, C_{2})$ depends on the Mooney-Rivlin coefficients $C_{i}$ and $F_{2} = F_{2}(C_{3}, C_{4}, C_{5}, \lambda ^*)$ depends on the exponential stress coefficient $C_{3}$, the rate of collagen uncrimping $C_{4}$, the elastic modulus of the straightened collagen fibres $C_{5}$, and the fibre stretch $\lambda ^*$ of straightened fibres. As before, $J$ denotes the determinant of the deformation gradient tensor and $k$ the bulk modulus. The material constants used are shown in Table III.

**TABLE III:** Material Parameters [MPa] for Ligaments in a Stress-Free State, as Well as Initial In-Situ Stretch [%] At Full Extension, cf. [27]

	$C_{1}$	$C_{2}$	$C_{3}$	$C_{4}$	$C_{5}$	$\lambda ^*$	$k$	$\lambda _{0}$
ACL	1.95	0.0	0.0139	116.22	535.039	1.046	73.2	1.07
PCL	3.25	0.0	0.1196	87.178	431.063	1.035	122	0.0
MCL	1.44	0.0	0.57	48.0	467.1	1.063	70	1.04
LCL	1.44	0.0	0.57	48.0	467.1	1.063	70	1.03

*Pre-strain:* Since the geometries are taken from in-vivo data, they are subjected to loads in the reference configuration. Using the Prestrain plugin [19] of FEBio [18], the analysis can be started from such a pre-stressed ligament configuration. The pre-stress can be defined by the hyperelastic constitutive formulation. We assume that the total deformation gradient tensor corresponding to the current state $F_{e}$ is obtained by the composite deformation

\begin{equation*}
F_{e} =F\, F_{p}, \tag{4}
\end{equation*}

where $F_{p}$ represents the deformation gradient according to the initial pre-strain state and $F$ results from external loads to the stress-free reference configuration. The pre-strain gradient tensor $F_{p}$ can be calculated based on the initial in-situ stretch $\lambda _{0}$ and the fibre vector defined by the elastic material. The initial strains in the present model are included in Table III.

### C. Contact and Boundary Conditions

In the described FE model, mesh nodes that fulfill given conditions are automatically detected and written into the simulation control file. For contact conditions of ligaments attached to bone at their proximal and/or distal ends, all ligament mesh nodes in their intersection to the corresponding bone, so-called footprints, are detected. In FEBio, the footprints need to be defined to match the rigid body motion of the attached bone. These footprints are acquired through the intersection of the segmentation masks of the ligament and the respective bone. Similarly, the mesh nodes in the contact area of cartilages and bones are prescribed to have the same motion as the associated moving part. Frictionless non-linear contact conditions allow structures to slide across each other without penetration. 20 potential contact zones have been identified: between each bone and ligament, femoral cartilage contacts to tibial cartilage, medial and lateral meniscus to the associated cartilage area, as well as contact at the cruciate ligaments and occasional contact between menisci and ligament.

**Fig. 4.**
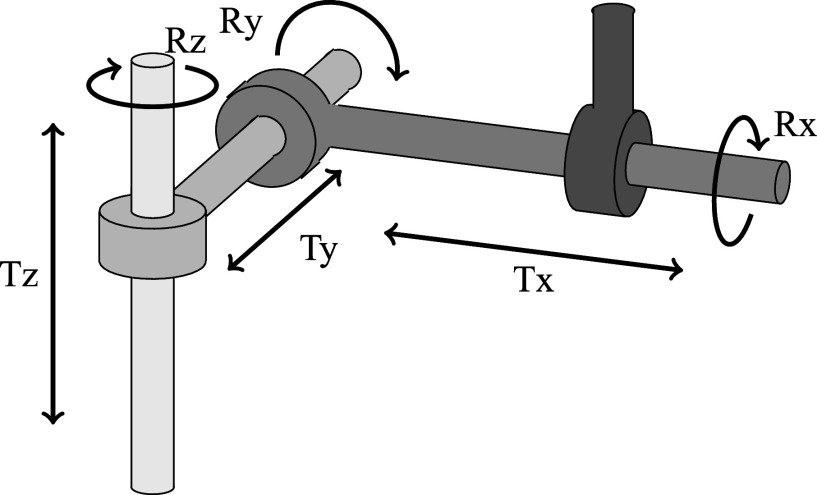
The joint system is modeled as a four-link kinematic chain consisting of cylindrical joints.

The motion of each bone can be addressed by the six degrees of freedom. In all analyses, proximal tibia and fibula remained fixed. In order to align the motion of flexion and rotation from the measured MR data in various knee positions, an image-based registration has been performed with a masked similarity metric. For the FE simulation, a direct or indirect motion model can be applied. An individual, interpolated motion can be prescribed directly as boundary conditions to the femur at all times. Through this direct motion model, one can analyse the resulting stress and pressure distribution in the ligaments, cartilages and menisci. On the other hand, the indirect motion model uses a cylinder system (cf. Fig. 4), first developed by [30], to model the motion in the knee joint. Here, only few degrees of freedom need to be specified, e.g., define the flexion angle Rx to model flexion. The resulting motion is uniquely defined owing to the anatomical arrangement of the structures in the knee. This indirect model is the preferred approach in the present investigations and is used to validate the FE model in Section III-B.

### D. Acquiring and Processing Ground Truth MR Images

**Fig. 5.**
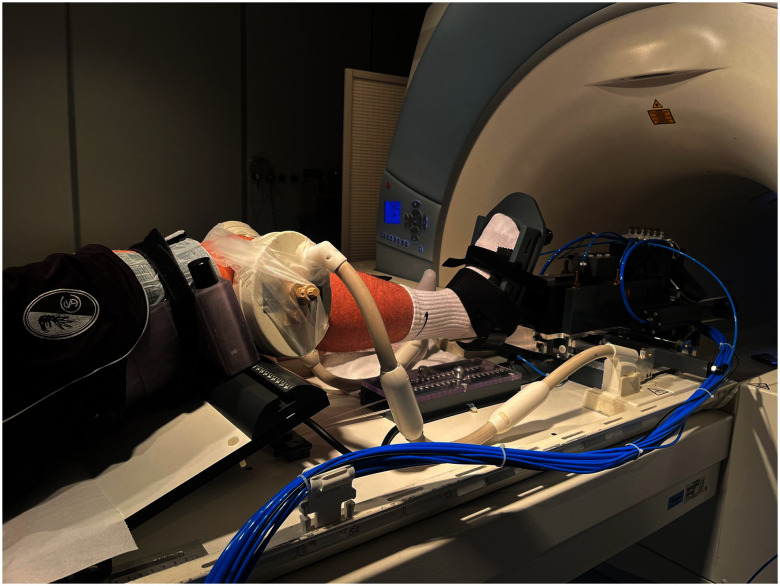
Volunteer placed in the pneumatic loading device for ground truth MR image acquisition.

**Fig. 6.**
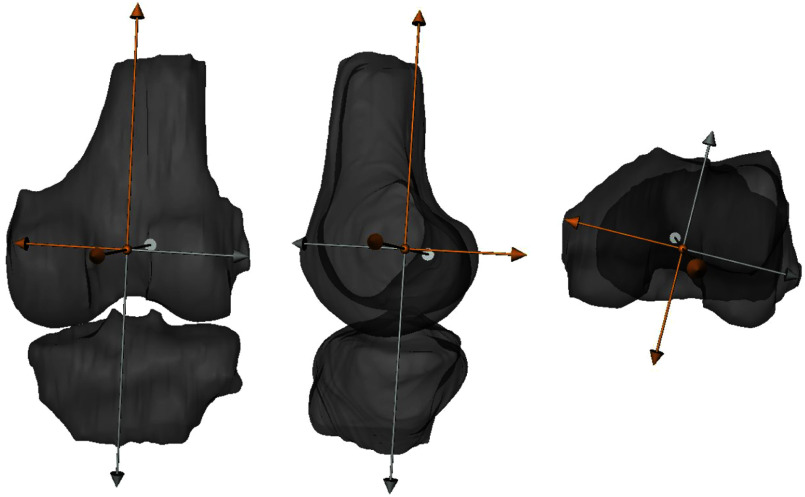
Illustration of the femur coordinate system for the right knee of volunteer V19, posterior, lateral, and top view (from left to right).

For ground truth image acquisition, a Magnetom Trio 3 T system (Siemens Healthineers, Germany) with an 8-channel multipurpose coil (NORAS MRI products, Germany) was used. All MR scans were performed with a T1-weighted spoiled 3D gradient-echo sequence using slab-selective water excitation and covering a FOV of 145 mm (AP) × 125 mm (RL) × 160 mm (FH) with a spatial resolution of 0.6 mm (AP) × 0.5 mm (RL) × 0.6 mm (FH). Further sequence parameters were: TR = 16 ms, TE = 6.88 ms, exc. angle = 15$^\circ$, readout bandwidth = 130 Hz/Px, readout direction = AP. The MR scan duration was 5:34 min. To mitigate motion artifacts and ensure acceptable image quality, the MR imaging sequence was augmented with prospective motion correction based on a moiré phase tracking system (Metria Innovation Inc., Milwaukee, US) [17], [31], [32]. The tracking system consisted of a single in-bore camera and a single tracking marker, which created angle-dependent moiré patterns for accurate detection of rotational motion. The tracking marker was attached to the center of the knee cap. Motion was tracked in six degrees of freedom with a frame rate of 80 frames/s. A position update of the MR measurement volume was performed before every excitation pulse. For the MR scans, the examined leg was placed in a MR-compatible pneumatic device, which enabled the knee to be brought into the stress positions of interest by applying defined rotational forces to the knee joint, see Fig. 5. Volunteers were placed in the device and individual adjustments were made depending on height and body proportions. Correct foot and femoral fixation were ensured. The device was controlled from the console room. It achieved 20$^\circ$ flexion by elevating the thigh via a pneumatic pillow and retracting the foot piece to maintain heel contact. Scans with 5 Nm internal/external rotation torque applied to the foot were performed. A fully automatic surface-based Active Appearance Model according to [33] was build and used for segmenting the proximal tibia and distal femur bones in these ground truth Trio MR images. From this automatically created segmentation, voxel masks are created and used for registering the Trio ground truth and the Prisma model data. For describing the knee joint motion, we introduce an anatomical femur coordinate system, see Fig. 6.

The anatomical axes are estimated based on the segmentation mask of the femur, where the femoral and transepicondylar axis are extracted using image analysis and the AP axis is computed as dot-product of the latter. The femoral axis is corrected by a Gram-Schmidt step to generate an orthogonal axis system. We measure any motion with respect to this volunteer-specific coordinate system. The motion states (angles and translations) computed from the Trio MR images are summarized in Table IV. Note that the orientation of the rotation (positive/negative) for the Ry and Rz component differs depending on the laterality of the volunteer's knee, because a right-handed coordinate system was used independent of the laterality.

**TABLE IV:** Laterality (R for Right, L for Left) and Reference Angular and Translational Components of Motion States Computed From Static Ground Truth Trio MR Images as Well as Their Mean and Standard Deviation (STD)

	V01	V02	V05	V10	V15	V16	V18	V19	V23	V24	V29	mean	STD
Laterality	R	L	L	R	R	L	L	R	L	R	L	-	-
Rx 0$^\circ$ [$^\circ$]	4.2	11.1	8.2	4.7	6.1	14.6	6.9	9.6	8.7	12.5	6.5	8.5	3.3
Ry 0$^\circ$ [$^\circ$]	-0.3	0.0	0.8	-0.2	0.2	3.1	1.2	1.0	0.4	-1.0	0.2	0.5	1.1
Rz 0$^\circ$ [$^\circ$]	2.6	-10.0	-10.0	6.0	7.1	-6.2	-10.8	9.3	-4.5	1.9	-1.3	-1.4	7.3
Tx 0$^\circ$ [mm]	0.0	-1.6	-1.0	0.3	0.7	-0.1	-0.9	1.4	-0.4	0.6	-0.3	-0.1	0.9
Ty 0$^\circ$ [mm]	-1.2	-4.1	-3.3	-0.8	-1.6	-5.2	-2.7	-2.1	-2.5	-2.9	-1.9	-2.6	1.3
Tz 0$^\circ$ [mm]	-0.1	0.3	-0.4	0.2	0.0	0.0	-0.4	-0.1	0.8	-0.6	0.0	-0.0	0.4
Rx 20$^\circ$ [$^\circ$]	-18.2	-11.2	-13.0	-17.5	-28.3	-14.2	-13.2	-14.7	-17.7	-21.3	-23.3	-17.5	5.1
Ry 20$^\circ$ [$^\circ$]	1.2	0.6	-1.1	-0.3	0.5	-0.4	1.1	0.2	-1.5	-0.3	-0.9	-0.1	0.9
Rz 20$^\circ$ [$^\circ$]	-0.7	-9.9	3.6	1.7	-8.9	-10.8	-8.1	1.5	-1.0	4.0	3.6	-2.3	5.9
Tx 20$^\circ$ [mm]	-1.1	-1.3	0.5	-1.1	-1.2	0.7	-1.1	-0.9	1.1	0.0	0.9	-0.3	1.0
Ty 20$^\circ$ [mm]	2.5	-5.0	3.7	2.1	5.0	1.9	-2.0	-1.3	-0.5	-2.5	0.7	0.4	3.0
Tz 20$^\circ$ [mm]	-0.4	0.6	0.7	0.2	0.4	-0.4	0.2	0.4	-0.6	1.3	0.8	0.3	0.6
Rx 20$^\circ$+ER [$^\circ$]	-16.9	-12.0	-12.8	-20.0	-25.5	-12.0	-19.5	-14.9	-19.0	-19.0	-22.6	-17.7	4.4
Ry 20$^\circ$+ER [$^\circ$]	0.3	3.1	1.6	-3.0	2.0	1.6	3.3	-1.7	-0.4	-3.2	0.2	0.3	2.3
Rz 20$^\circ$+ER [$^\circ$]	11.8	-24.7	-13.7	22.1	23.5	-22.5	-21.7	21.4	-13.7	22.1	-9.8	-0.5	20.5
Tx 20$^\circ$+ER [mm]	-1.5	0.3	2.1	-1.9	-1.0	2.0	0.0	-0.6	1.7	0.8	0.9	0.3	1.4
Ty 20$^\circ$+ER [mm]	3.8	0.5	2.7	4.6	4.5	4.9	1.5	0.7	0.7	-0.9	1.6	2.2	2.0
Tz 20$^\circ$+ER [mm]	0.2	1.7	1.5	0.7	0.3	0.0	0.5	1.6	-0.5	1.1	1.0	0.7	0.7
Rx 20$^\circ$+IR [$^\circ$]	-19.5	-12.6	-14.5	-18.0	-30.0	-14.5	-14.1	-17.9	-18.6	-20.5	-24.2	-18.6	5.1
Ry 20$^\circ$+IR [$^\circ$]	1.3	-0.3	-1.6	1.1	-1.2	-5.0	-0.9	0.0	-2.0	0.9	-1.0	-0.8	1.8
Rz 20$^\circ$+IR [$^\circ$]	-10.2	5.0	12.5	-14.6	-18.9	15.2	17.0	-11.8	8.2	-11.7	12.5	0.3	13.7
Tx 20$^\circ$+IR [mm]	-0.5	0.2	-0.1	-0.6	-0.7	-2.0	0.4	-1.0	0.2	-1.4	0.7	-0.4	0.8
Ty 20$^\circ$+IR [mm]	3.7	-0.3	3.8	4.1	7.0	2.7	2.7	2.7	3.1	-0.3	3.1	2.9	2.0
Tz 20$^\circ$+IR [mm]	-0.3	0.3	1.0	0.8	0.0	-0.1	1.0	0.7	-0.6	1.9	1.0	0.5	0.7

## III. Results

In this section, we provide results of the FE simulation for the eleven volunteers of the study described in Section II-D. We compare FE model predictions against the ground truth Trio MR measurements for the knee in extension (0$^\circ$), in 20$^\circ$ flexion (20$^\circ$), and in internal and external rotation at 20$^\circ$ flexion (20$^\circ$+IR, 20$^\circ$+ER).

### A. FE Models

The FE meshes were automatically generated by TetGen [23]. The number of nodes and elements of each voluneer's meshes are summarized in Table V. Simulations were performed on an Intel(R) Xeon(R) CPU with 2.10 GHz using two cores. We simulated two motion sequences starting from model configuration via 0$^\circ$ flexion and 20$^\circ$ flexion to internal or external rotation motion poses. We will refer to these motion sequences as 0$^\circ$-20$^\circ$-IR and 0$^\circ$-20$^\circ$-ER. The average computation time in direct motion mode was 2 hours per motion sequence. Using the indirect motion mode prescribing the flexion (Rx) and deformity (Ry) angle, leads to significantly higher computation times, see Table V.

**TABLE V:** Number of nodes/elements of the Generated FE Models and Runtime for the Motion Sequences 0$^\circ$-20$^\circ$-IR (Runtime Internal Rotation (IR)) and 0$^\circ$-20$^\circ$-ER (Runtime External Rotation (ER))

volunteer	# nodes	# elements	runtime IR [h]	runtime ER [h]
V01	7050	20896	17	11
V02	7041	20125	28	32
V05	7268	21873	19	13
V10	3984	9701	14	14
V15	5821	16596	19	20
V16	6021	17120	24	17
V18	5587	16072	14	15
V19	8438	24211	21	24
V23	7453	22359	17	27
V24	6027	18047	16	17
V29	6260	17687	22	21

### B. Validation of the FE Model

**Fig. 7.**
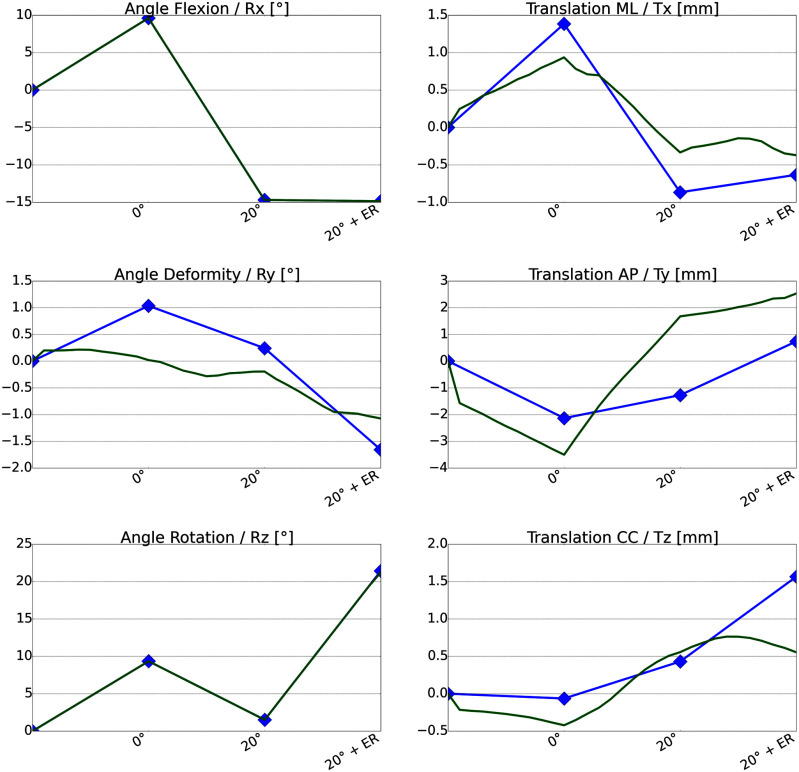
Motion curves of the femoral bone of volunteer V19 for the 0$^\circ$-20$^\circ$-ER motion sequence for FEM simulation (green) and the ground truth Trio MR (blue) simulation.

**Fig. 8.**
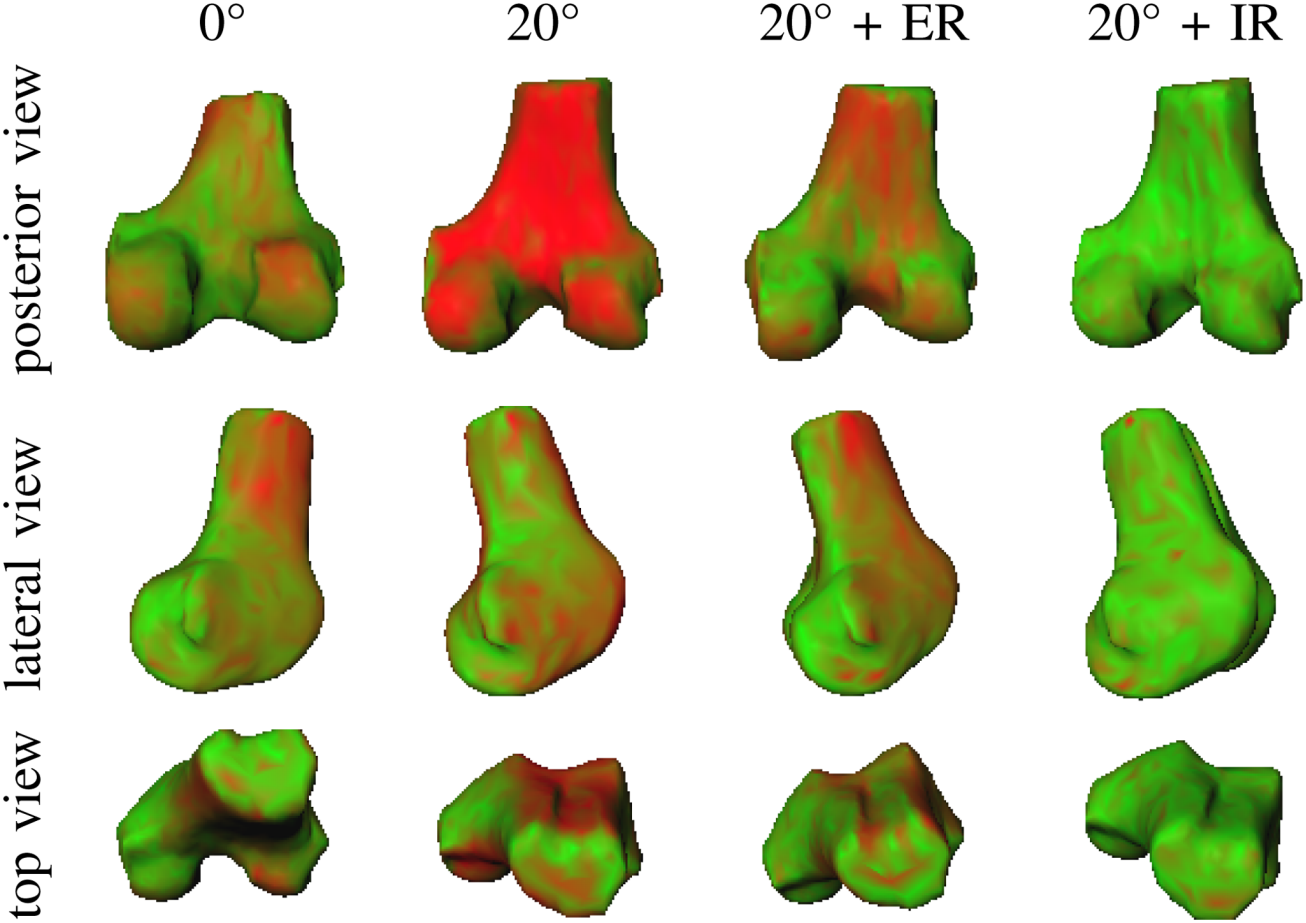
Difference between the MR-based and the FE-based femur poses for 0$^\circ$ flexion, 20$^\circ$ flexion, and additional external (20$^\circ$ + ER) and internal (20$^\circ$ + IR) rotation motion states of volunteer V19; green color: 0 mm deviation, red color: 3 mm deviation.

**Fig. 9.**
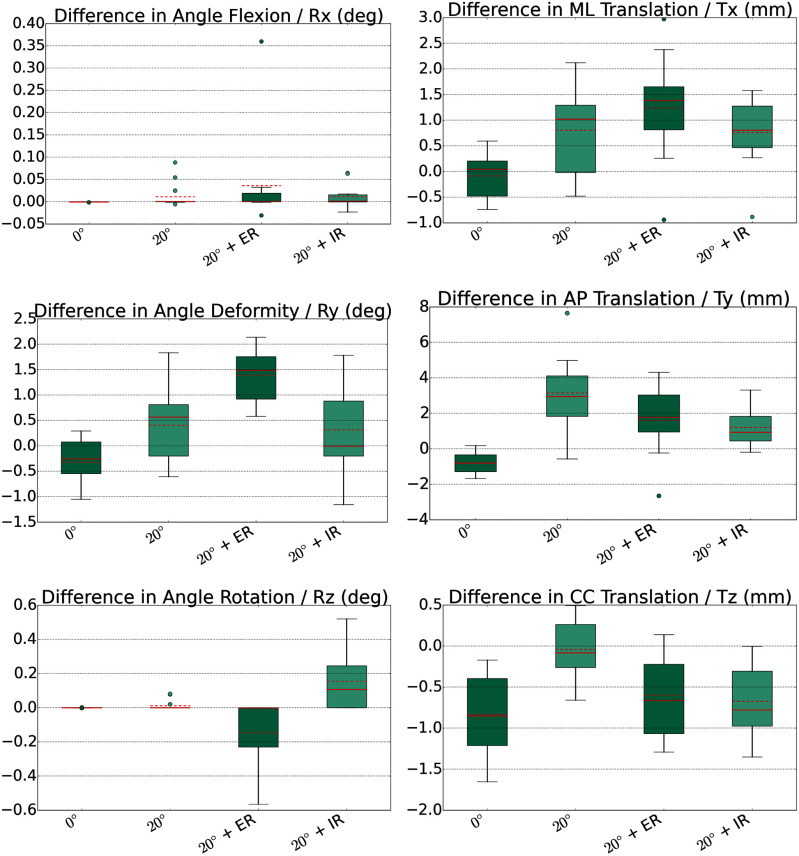
Rotational and translational differences between FE-based and MR-based femur poses across all eleven volunteers.

We derived the motion of the femur relative to the tibia and fibula from the ground truth Trio MR data by rigid image registration. Rigid registration restricted to the tibial bone mask was applied to initially align a pair of images. Rigid registration restricted to the femoral bone mask then gives an according rigid motion of the femur, which can be described by an affine matrix. We use (spherical) linear interpolation to retrieve a reference motion. Fig. 7 exemplarily shows the overlay of measured MR and simulated FE motion curves for a 0$^\circ$-20$^\circ$-ER motion sequence for the volunteer V19. The 3D motion of the femur is represented by its rotation (Rx, Ry, Rz) in degree around the medial-lateral (ML), AP and carniocaudal (CC) anatomical femur coordinate axes and the translations (Tx, Ty, Tz) in millimeters along these axes, see II-D. The motion curves for the ground truth Trio MR data and the FE motion of V19 are only a few millimetres or degrees apart with a maximum of 2.96 mm difference in AP translation for the 20$^\circ$ flexion state (volunteer average: 3.16 mm) and a maximum of 0.58$^\circ$ difference in Ry for the external rotation (volunteer average: 1.38$^\circ$). Fig. 8 depicts the differences of the FE-based and the MR-based femur pose for 0$^\circ$/20$^\circ$ flexion and internal/external rotation of V19. The colour code is quantitative: green corresponds to 0 mm and red to 3 mm Hausdorff surface distance. We summarize the differences in the rotation angles and translations for all volunteers in the box plots shown in Fig. 9. These plots reconfirm the good consistency between the ground truth Trio data and the FE-based simulation. In alignment with our previous findings we observe differences between simulation and MR data of less than 1.7 mm for all motion states with an exception of 3.16 mm average AP translation in the 20$^\circ$ flexion position. For the rotation angles we observe differences less than 0.5$^\circ$ for all motion states with an exception of 1.38$^\circ$ average difference in the deformity angle for the external rotation.

### C. Analysis of Ligament and Menisci Stress During Flexion, Internal and External Rotation

**Fig. 10.**
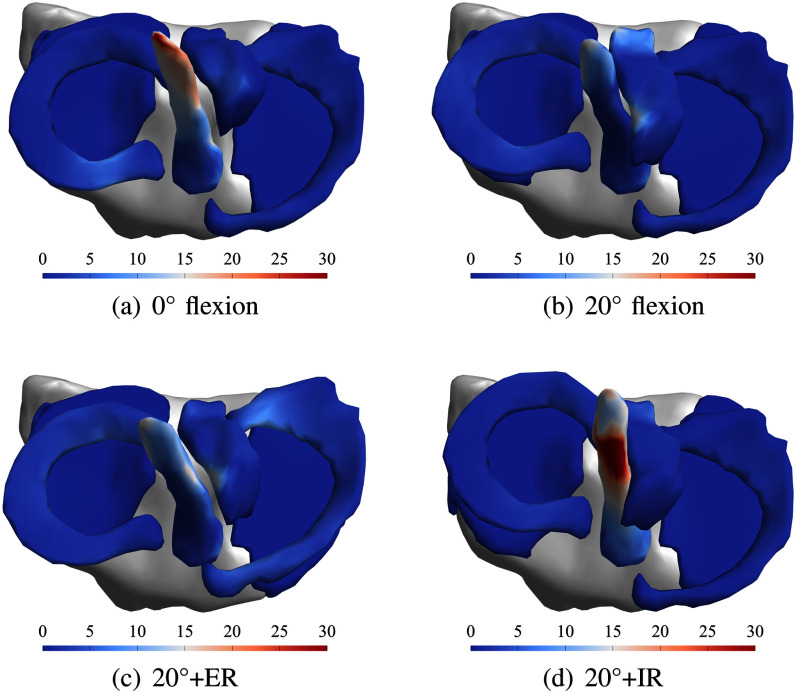
Effective/von Mises stresses [MPa] in ACL, PCL and menisci for volunteer V19.

The effective/von Mises stresses [34] in the ACL, PCL, medial and lateral menisci computed by the FE simulation of the right knee of volunteer V19 in 0$^\circ$/20$^\circ$ flexion and external/internal rotation motion states are shown in Fig. 10. We observe a nearly stress-free and balanced state in the 20$^\circ$ position (top right), while the ACL shows medium stress in the extended knee (0$^\circ$ position, top left). External rotation (bottom left) introduces stress in the lateral meniscus (light blue and white regions) and low to medium stress in both ACL and PCL. Internal rotation (bottom right) introduces high stress in the ACL, while the PCL and menisci stay in a nearly stress-free condition. Highest stress is observed in the ACL during internal rotation.

## IV. Discussion

We presented a FE model that is amenable to predict the knee joint kinematics from static MR images. We validated the model with in-vivo measurements using a loading device within the MR scanner to image the knee in loaded positions like internal/external rotation. The model is able to predict these states with an average accuracy of approximately 2 mm in the translational components and 1$^\circ$ for the rotational components, see Fig. 9 for details. Tunnel misplacement has to be considered the major course for graft failure in anterior cruciate ligament injury. Interestingly, surgical precision for tunnel placement is low. Deviations from planned to placed tunnel positions are in the range of 5-10 mm at the tibia and femur respectively. However from a clinical perspective an accuracy of $< \!\! $ 2 mm for tunnel placement and prediction of translation is desirable. For rotatory stabilization there exists little knowledge about the inter- and intra-individual differences and required accuracy for predictions.

However, the measurement setup still has limitations. First, the procedure of positioning a volunteer is not fully reproducable at high accuracy due to minor positioning flexibility in the hip/knee joint and in the foot piece. The exact position and motion history influence the internal geometric state of the knee joint in the respective measured positions. Furthermore, especially in 20$^\circ$ flexion, the ligaments may reach relaxed states as discussed in Section III-C. The knee joint position is then no longer clearly defined by the ligaments, but by the positioning and gravity, which we neglected in our model.

To simplify the model boundary conditions, the patella and the patella/quadriceps tendons have been neglected. Incorporating these structures into the model as well as analyzing the patella pose accuracy in the predictions is under current investigation.

There are several topics to be investigated in future work. Our model is based on average material parameters taken from literature and does not incorporate individual parameter adaptations. However, individual material parameters could be estimated, e.g., with the measurement data currently used for validation, to increase the model prediction accuracy. Furthermore, we also plan to incorporate soft tissue structures (e.g. menisci) from the ground truth MR images in the validation of the simulation model. Moreover, including active muscle contraction into the presented framework might increase the accuracy, especially in loaded configurations, but also comes at high computational costs, mainly due to the need to model structures of the whole leg. The study described in Section II will also be conducted on patients suffering from ACL rupture, and will contain both, pre- and post-operative measurements. Evaluation of these data and of the accuracy of the presented FE model applied to these cases will provide insights how the presented model can be used in the decision process for patient treatment, e.g. by suggestion optimal positions for an ACL graft. During clinical testing the so called “Pivot-Shift-Test” investigates joint stability during the transition from extension to low flexion during valgus stress and internal rotation. It is the best test for assessment of postoperative stability and functional clinical outcome. However, so far it is not possible to clinically standardize the test and quantify stability during clinical routine. The developed knee model can predict translational and rotational stability during stance and low flexion. Moreover the model could be used to place grafts more according to the individual needs of the patient, i.e. predominantly translational stability.

In order to fully automate the model generation process described in Section II, we are currently developing automatic segmentation tools for bones, cartilages, menisci, and ligaments, thereby providing accurate segmentation within a few minutes and reducing errors and variability in manual segmentation. Nevertheless, we observed consistently predicted kinematics from independent manual segmentation from the same imaging data.

## V. Conclusion

Our vision is to provide clinicians with data of the individual knee kinematics to support the decision making process, to improve the patient's treatment, and to restore physiological joint kinematics in an injured knee. Our main contribution to this vision is the validation of the FE simulation approach for predicting the individual knee joint kinematics using in-vivo data. The validation was based on static MR images of the knee in different loaded positions. These knee joint positions were controlled using a MR-compatible pneumatic loading device. The presented individual FE simulations are able to predict the individual kinematics with clinical sufficient accuracy. Thus, our approach can be used in the decision-making process to restore knee joint stability and functionality after knee injuries, e.g. ACL ruptures. Future applications would in addition be able to standardize postoperative rehabilitation and injury prevention as exercises with low local stress to injured structures could be suggested.
